# Association of food intake with a risk of metabolic dysfunction-associated fatty liver disease: a cross-sectional study

**DOI:** 10.1093/gastro/goad054

**Published:** 2023-09-11

**Authors:** Xian-Hua Huang, He-Wei Peng, Jing-Ru Huang, Rong Yu, Zhi-Jian Hu, Xian-E Peng

**Affiliations:** Department of Epidemiology and Health Statistics, Fujian Provincial Key Laboratory of Environment Factors and Cancer, School of Public Health, Fujian Medical University, Fuzhou, Fujian, P. R. China; Department of Epidemiology and Health Statistics, Fujian Provincial Key Laboratory of Environment Factors and Cancer, School of Public Health, Fujian Medical University, Fuzhou, Fujian, P. R. China; College of Integrated Chinese and Western Medicine, Fujian University of Traditional Chinese Medicine, Fuzhou, Fujian, P. R. China; Department of Epidemiology and Health Statistics, Fujian Provincial Key Laboratory of Environment Factors and Cancer, School of Public Health, Fujian Medical University, Fuzhou, Fujian, P. R. China; Department of Epidemiology and Health Statistics, Fujian Provincial Key Laboratory of Environment Factors and Cancer, School of Public Health, Fujian Medical University, Fuzhou, Fujian, P. R. China; Department of Epidemiology and Health Statistics, Fujian Provincial Key Laboratory of Environment Factors and Cancer, School of Public Health, Fujian Medical University, Fuzhou, Fujian, P. R. China; Key Laboratory of Gastrointestinal Cancer, Ministry of Education, Fujian Medical University, Fuzhou, Fujian, P. R. China

**Keywords:** metabolic dysfunction-associated fatty liver disease, food intake, cross-sectional study, inverse probability of treatment weighting

## Abstract

**Background:**

Metabolic dysfunction-associated fatty liver disease (MAFLD) is a common liver disease, the risk of which can be increased by poor diet. The objective of this study was to evaluate the associations between food items and MAFLD, and to propose reasonable dietary recommendations for the prevention of MAFLD.

**Methods:**

Physical examination data were collected from April 2015 through August 2017 at Nanping First Hospital (*n *=* *3,563). Dietary intakes were assessed using a semi-quantitative food frequency questionnaire. The association between food intake and the risk of MAFLD was assessed by using the inverse probability weighted propensity score.

**Results:**

Beverages (soft drinks and sugar-sweetened beverages) and instant noodles were positively associated with MAFLD risk, adjusting for smoking, drinking, tea intake, and weekly hours of physical activity [adjusted odds ratio (OR_adjusted_): 1.568; *P *=* *0.044; OR_adjusted_: 4.363; *P *=* *0.001]. Milk, tubers, and vegetables were negatively associated with MAFLD risk (OR_adjusted_: 0.912; *P *=* *0.002; OR_adjusted_: 0.633; *P *=* *0.007; OR_adjusted_: 0.962; *P *=* *0.028). In subgroup analysis, the results showed that women [odds ratio (OR): 0.341, 95% confidence interval (CI): 0.172–0.676] had a significantly lower risk of MAFLD through consuming more tubers than men (OR: 0.732, 95% CI: 0.564–0.951).

**Conclusions:**

These findings suggest that reducing consumption of beverages (soft drinks and sugar-sweetened beverages) and instant noodles, and consuming more milk, vegetables, and tubers may reduce the risk of MAFLD.

## Introduction

Non-alcoholic fatty liver disease (NAFLD) is the most common chronic liver disease, with an estimated prevalence of 25% worldwide [1]. In Asia, China has the highest NAFLD mortality, morbidity, and annual mortality rates [2]. Recently, an international panel of hepatologists proposed renaming NAFLD as metabolic dysfunction-associated fatty liver disease (MAFLD) [3, 4]. The diagnostic criteria for MAFLD differ from those of NAFLD [3, 5]. Specifically, MAFLD diagnosis requires the presence of hepatic steatosis confirmed by histological biopsy, imaging, or blood biomarkers, in addition to at least one of three criteria: overweight/obesity, type 2 diabetes mellitus, or metabolic disorders. The updated definition of MAFLD, which incorporates new diagnostic criteria for other fatty liver diseases, enables more accurate identification of patients with fatty liver who are highly susceptible to disease progression [6, 7]. The current research on liver diseases based on imaging has been proved to be helpful for the early diagnosis and treatment of MAFLD [8], and we thought the research on the risk factors related to MAFLD may be crucial for the prevention and control of diseases. By understanding the underlying causes and identifying high-risk populations, effective prevention and treatment strategies can be developed to mitigate the impact of these conditions.

Many studies have indicated that MAFLD results from a combination of multiple factors and mechanisms [9, 10]. Diet may affect the occurrence and development of MAFLD [11–13]. For example, high energy intake leads to the accumulation of triglycerides in adipose tissue and liver. The Westernized diet characterized by high fat, high sugar, high meat, and low plant fiber consumption has been implicated as a risk factor for MAFLD by several studies [14, 15]. However, current research on the correlation between foods and MAFLD is rare, and further research is needed. Thus, the purpose of this study was to investigate the association of food items with MAFLD and propose reasonable dietary recommendations for the prevention of MAFLD.

## Methods

### Study design and subjects

**Figure 1.**
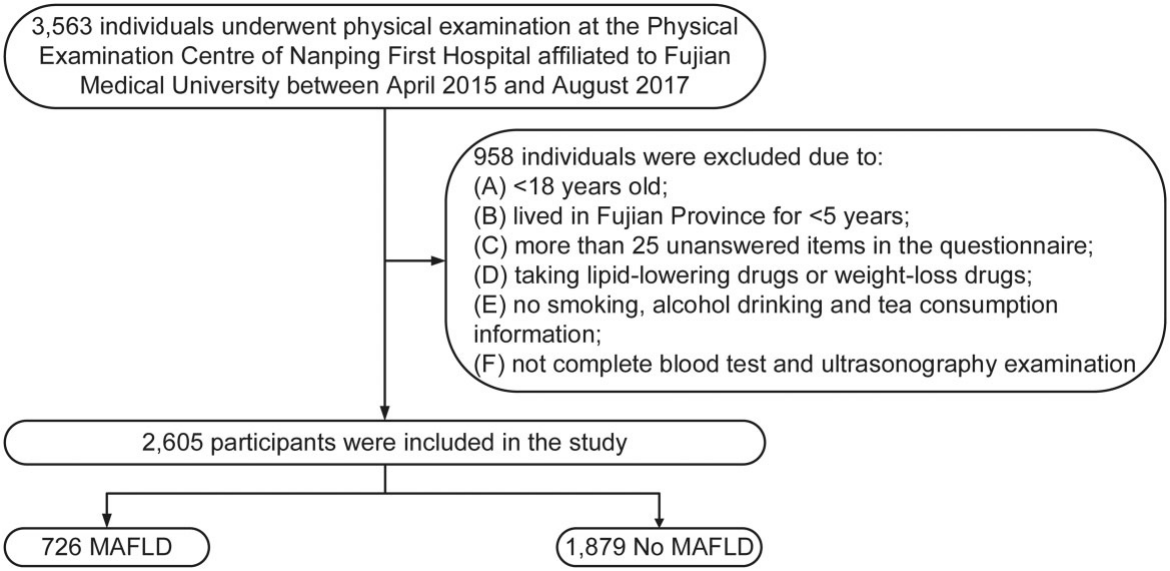
Flow chart of study population. MAFLD, metabolic dysfunction-associated fatty liver disease. No MAFLD, non-metabolic dysfunction-associated fatty liver disease.

The study was of a cross-sectional survey design, involving a population of people who underwent physical examination at the Physical Examination Centre of Nanping First Hospital Affiliated to Fujian Medical University (Nanping, Fujian, China) from April 2015 to August 2017. As shown in Figure 1, in accordance with the exclusion criteria, a portion of participants were excluded from this study, including: (i) participants who were <18 years old; (ii) residents who had lived in Fujian province for <5 years; (iii) participants who had >25 unanswered items in the questionnaire; (iv) participants who were taking lipid-lowering drugs or weight-loss drugs; (v) people who did not provide information on smoking, alcohol drinking, and tea consumption; (vi) participants who did not complete blood test and ultrasonography examination. All subjects provided written informed consent before they participated in this study.

### Data collection

#### MAFLD ascertainment

MAFLD was diagnosed by using ultrasound showing hepatic steatosis and having one of the following three criteria: overweight or obesity [body mass index (BMI) ≥ 23 kg/m^2^], type 2 diabetes mellitus, and the presence of at least two risk factors for metabolic abnormalities: (i) waist circumference of ≥90 cm for men and ≥80 cm for women; (ii) systolic blood pressure of >130 mmHg or diastolic blood pressure of >85 mmHg or taking antihypertensive drugs; (iii) plasma triglyceride of ≥1.7 mmol/L or taking specific anti-lipid agents; (iv) high-density lipoprotein levels of <1.0 mmol/L for men and <1.3 mmol/L for women or taking high-density lipoprotein (HDL)-regulating drugs; (v) prediabetes—fasting plasma glucose level of 5.6–6.9 mmol/L, 2-hour post-load glucose levels of 7.8–11.0 mmol, or HbA1c 5.7%–6.4%; (vi) homeostasis model assessment for insulin resistance index of ≥2.5; (vii) plasma hypersensitive C-reactive protein of ≥2.0 mg/L [3]. Hepatic ultrasonography was performed by experienced radiologists who did not have access to any relevant clinical data and diagnosis.

#### Dietary information collection

A semi-quantitative food frequency questionnaire containing 110 food items was used to collect information about participants’ typical food consumption, which was developed and validated in a sample from southern China [16]. For each individual food item, participants were asked to report their average frequency of the consumption during the past year. The frequency of consumption was divided into eight categories: 3 times/day, 2 times/day, 1 times/day, 1–2 times/week, 3–4 times/week, 5–6 times/week, 1–3 times/month, and <once/month. Red meat included pork, beef, lamb, and offal. Poultry included chicken and duck. Aquatic products were defined as fish, crab, and shellfish. Beverages include soft drinks and sugar-sweetened beverages.

#### Evaluation and definition of other variables

The information included age, sex, marital status, income, education level, smoking status, tea consumption, physical activity, drug use, and medical status. All patients had a physical examination (height, weight, waist circumference, hip circumference, and blood pressure) and blood samples were obtained (fasting plasma glucose, low-density lipoprotein (LDL), HDL, total cholesterol, triglycerides, alanine aminotransferase, aspartate aminotransferase, and γ-glutamyl transferase).

The BMI was calculated as weight/height^2^ (kg/m^2^). Hypertension was defined as systolic blood pressure of >140 mmHg or diastolic blood pressure of >90 mmHg [17]. Type 2 diabetes mellitus was diagnosed as fasting plasma glucose of ≥7.0 mmol/L or 2-hour post-load glucose levels of ≥11.1 mmol/L [18].

### Statistical analysis

We compared baseline characteristics by using the chi-square test for categorical characteristics, and *t*-test for continuous variables. Quantitative variables are presented as means ± standard deviation (SD). Categorical variables are presented as percentage. The propensity score weighted univariable and multivariable analysis can effectively control for potential differences in baseline characteristics of the included patients [19, 20]. Age, gender, and marital status were used to calculate the propensity score. And the inverse probability of treatment weighting was used to evaluate the correlation between smoked food, baked food, preserved food, beans, eggs, nuts, beverages (soft drinks and sugar-sweetened beverages), confectionery, fruits, fried food, cereals, fresh milk, potatoes, vegetables, instant noodles, red meat, aquatic products, poultry, and MAFLD, adjusting for smoking status, drinking status, tea intake status, and weekly hours of physical activity.

We also conducted subgroup analysis to examine the relationships of milk and tubers with MAFLD by the following subgroups: age (<45 years or ≥45 years), gender (male or female), marital status (single or other, married), smoking status (yes or no), tea consumption status (yes or no), drinking status (yes or no), and weekly hours of physical activity (<14 h/week or ≥14 h/week). *P*-value for interaction was calculated.

SPSS19.0 (IBM SPSS, 2010, Chicago, IL, USA) was used for statistical analysis. All *P*-values were two-sided and *P *<* *0.05 was considered to represent a statistically significant result.

## Results

### Baseline characteristics

A total of 2,605 participants were included in the study. In the entire sample, 56.5% were male, the mean age was 43.17 ± 12.17 years, and the mean BMI was 23.10 ± 3.05 kg/m^2^. As shown in Table 1, compared with non-MAFLD, participants in the MAFLD group were found to be older, married, smokers, drinkers, tea drinkers, with a higher prevalence of diabetes and hypertension, and more likely to be male (all *P *<* *0.05). In addition, there were differences in BMI, hip circumference, waist circumference, weekly hours of physical activity, systolic blood pressure, diastolic blood pressure, γ-glutamyl transferase, alanine aminotransferase, aspartate aminotransferase, total cholesterol, total triglyceride, LDL, and HDL between the two groups (all *P *<* *0.05).

**Table 1.** Characteristics of the study population

Characteristic	MAFLD	No MAFLD	*P*
	(*n *=* *726)	(*n *=* *1,879)	
Age (years), mean ± SD	45.90 ± 11.69	42.12 ± 12.19	<0.001
Gender, *n* (%)			<0.001
Male	584 (80.44)	887 (47.21)	
Female	142 (19.56)	992 (52.79)	
BMI (kg/m^2^), mean ± SD	25.52 ± 2.67	22.16 ± 2.64	<0.001
Educational level, *n* (%)			0.118
Primary school and less than	50 (6.89)	127 (6.76)	
Junior middle and high school	276 (38.02)	636 (33.85)	
Junior college or above	400 (55.10)	1,116 (59.39)	
Income (yuan/month), *n* (%)			0.066
<2,000	35 (4.82)	112 (5.96)	
2,000–3,000	202 (27.82)	592 (31.51)	
>3,000	489 (67.36)	1,175 (62.53)	
Marital status, *n* (%)			<0.001
Married	652 (89.81)	1,557 (82.86)	
Single or other	74 (10.19)	322 (17.14)	
Hip circumference (cm), mean ± SD	99.82 ± 5.97	93.81 ± 5.69	<0.001
Waist circumference (cm), mean ± SD	90.58 ± 7.78	79.03 ± 8.61	<0.001
Smoking status, *n* (%)			<0.001
Never	448 (61.71)	1,468 (78.13)	
Former	45 (6.20)	68 (3.62)	
Current	233 (32.09)	343 (18.25)	
Tea intake status, *n* (%)			<0.001
Never	205 (28.24)	847 (45.08)	
Former	3 (0.41)	5 (0.27)	
Current	518 (71.35)	1,027 (54.66)	
Drinking status, *n* (%)			<0.001
Never	389 (53.58)	1,235 (65.73)	
Former	15 (2.07)	27 (1.44)	
Current	322 (44.35)	617 (32.84)	
Diabetes, *n* (%)			<0.001
Yes	76 (10.47)	70 (3.73)	
No	650 (89.53)	1,809 (96.27)	
Hypertension, *n* (%)			<0.001
Yes	247 (34.02)	280 (14.90)	
No	479 (65.98)	1,599 (85.10)	
SBP (mmHg), mean ± SD	126.86 ± 15.77	116.25 ± 14.94	<0.001
DBP (mmHg), mean ± SD	85.64 ± 10.17	77.80 ± 10.29	<0.001
GGT (U/L), mean ± SD	46.15 ± 39.63	26.10 ± 23.87	<0.001
ALT (U/L), mean ± SD	33.49 ± 23.43	20.27 ± 14.79	<0.001
AST (U/L), mean ± SD	26.43 ± 12.78	22.04 ± 10.60	<0.001
TC (mmol/L), mean ± SD	5.27 ± 0.98	5.03 ± 1.74	<0.001
TG (mmol/L), mean ± SD	2.32 ± 1.73	1.31 ± 1.80	<0.001
HDL (mmol/L), mean ± SD	1.19 ± 0.46	1.38 ± 2.19	<0.001
LDL (mmol/L), mean ± SD	3.40 ± 2.58	3.31 ± 3.37	<0.001
Physical activity (hours/week), mean ± SD	17.47 ± 17.05	23.36 ± 23.03	<0.001

BMI, body mass index; SBP, systolic blood pressure; DBP, diastolic blood pressure; GGT, γ-glutamyl transferase; ALT, alanine aminotransferase; AST, aspartate aminotransferase; TC, total cholesterol; TG, total triglyceride; LDL, low-density lipoprotein; HDL, high-density lipoprotein.

### Distribution of MAFLD in different BMI groups

**Figure 2.**
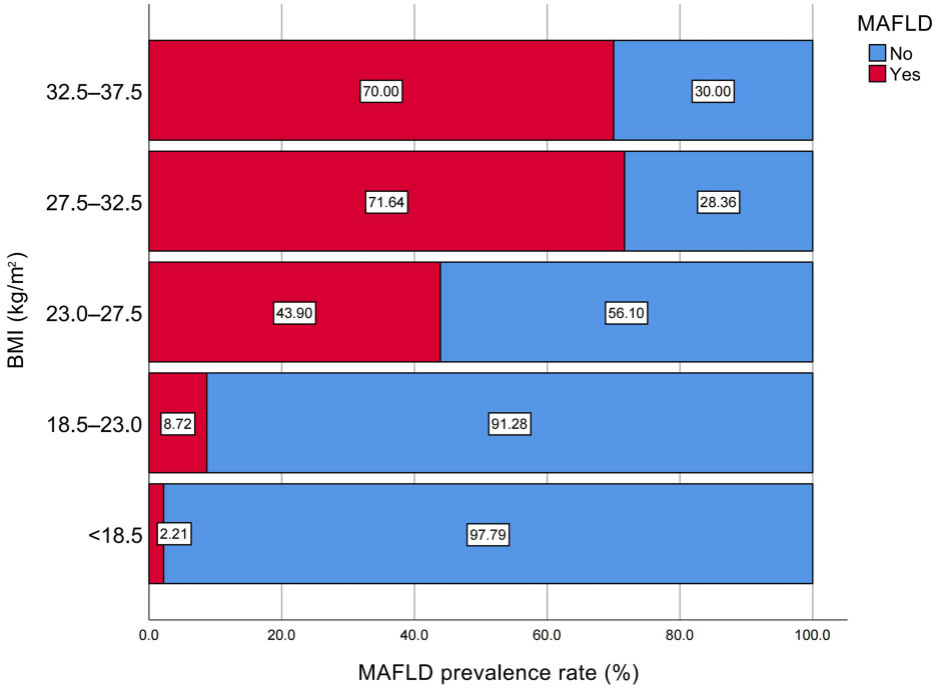
Metabolic Dysfunction-Associated Fatty Liver Disease (MAFLD) distribution in different Body Mass Index (BMI) groups.

The distribution of MAFLD in different BMI groups is shown in Figure 2. After grouping the study subjects according to BMI [21], it was found that there were significant differences in the distribution of MAFLD populations in different BMI levels (*P *<* *0.001) and the percentage of patients with MAFLD increased with the levels of BMI.

### Propensity score weighted univariable and multivariable analysis of associations between foods and MAFLD

As presented in Table 2, after propensity score weighting, milk, tubers, and vegetables were negatively correlated with the risk of MAFLD [milk odds ratio (OR): 0.919, 95% confidence interval (CI): 0.869–0.972; tubers OR: 0.628, 95% CI: 0.454–0.869; vegetables OR: 0.960, 95% CI: 0.928–0.994]. After adjustment for smoking status, drinking status, tea intake status, and weekly hours of physical activity, associations remained significant (milk OR: 0.912, 95% CI: 0.861–0.965; tubers OR: 0.633, 95% CI: 0.454–0.884; vegetables OR: 0.962, 95% CI: 0.930–0.996).

**Table 2.** Propensity score weighted univariable and multivariable analysis of associations between foods and MAFLD

Food species (per 50 g/day increase)	Crude OR (95% CI)	*P*	Adjusted OR (95% CI)*	*P*
Smoked foods	0.589 (0.193–1.801)	0.354	0.683 (0.215–2.163)	0.516
Baked goods	0.882 (0.663–1.175)	0.391	0.888 (0.657–1.200)	0.440
Pickled foods	0.990 (0.850–1.152)	0.892	0.984 (0.45–1.147)	0.840
Fried foods	1.443 (0.911–2.285)	0.119	1.460 (0.927–2.300)	0.102
Legumes	1.060 (0.844–1.331)	0.615	1.074 (0.853–1.353)	0.542
Egg	0.865 (0.673–1.113)	0.260	0.875 (0.679–1.129)	0.306
Nut	0.955 (0.884–1.032)	0.244	0.960 (0.888–1.037)	0.299
Beverage (soft drinks and sugar-sweetened beverages)	1.502 (0.976–2.310)	0.064	**1.568 (1.013–2.427)**	**0.044**
Candy	1.469 (0.588–3.668)	0.410	1.361 (0.542–3.416)	0.511
Fruit	0.972 (0.879–1.075)	0.581	0.972 (0.878–1.076)	0.583
Coarse cereals	0.984 (0.772–1.255)	0.897	0.981 (0.766–1.256)	0.887
Milk	**0.919 (0.869–0.972)**	**0.003**	**0.912 (0.861–0.965)**	**0.002**
Tubers	**0.628 (0.454–0.869)**	**0.005**	**0.633 (0.454–0.884)**	**0.007**
Vegetables	**0.960 (0.928–0.994)**	**0.020**	**0.962 (0.930–0.996)**	**0.028**
Instant noodles	**3.782 (1.575–9.081)**	**0.003**	**4.363 (1.789–10.642)**	**0.001**
Red meat	0.965 (0.879–1.059)	0.453	0.967 (0.880–1.062)	0.484
Seafood	1.003 (0.884–1.138)	0.964	1.009 (0.887–1.148)	0.888
Poultry	0.995 (0.762–1.298)	0.969	1.021 (0.780–1.337)	0.878

MAFLD, metabolic dysfunction-associated fatty liver disease.

* Adjusted by smoking status, drinking status, tea intake status, and weekly hours of physical activity.

Inversely, instant noodles were positively correlated with the risk of MAFLD (OR: 3.782, 95% CI: 1.575–9.081). Further adjustment for smoking status, drinking status, tea intake status, and weekly hours of physical activity, correlations of beverages (soft drinks and sugar-sweetened beverages) and instant noodles with MAFLD were statistically significant [beverages (soft drinks and sugar-sweetened beverages) OR: 1.568, 95% CI: 1.013–2.427; instant noodles OR: 4.363, 95% CI: 1.789–10.642].

### Sensitivity analyses

After adjustment for age, gender, marital status, smoking status, drinking status, tea intake status, and weekly hours of physical activity, milk, tubers, fruit, and beverages (soft drinks and sugar-sweetened beverages) were significantly associated with MAFLD [milk OR: 0.906, 95% CI: 0.861–0.953; tubers OR: 0.694, 95% CI: 0.542–0.888; fruit OR: 0.844, 95% CI: 0.772–0.922; beverages (soft drinks and sugar-sweetened beverages) OR: 1.571, 95% CI: 1.075–2.294] (Supplementary Table 1).

In addition, in propensity score matching analysis, milk, tubers, and instant noodles were significantly associated with MAFLD (milk OR: 0.866, 95% CI: 0.819–0.915; tubers OR: 0.762, 95% CI: 0.587–0.990; instant noodles OR: 3.962, 95% CI: 1.562–10.048), adjusted for smoking status, drinking status, tea intake status, and weekly hours of physical activity (Supplementary Table 2).

### Subgroup analysis

**Figure 3.**
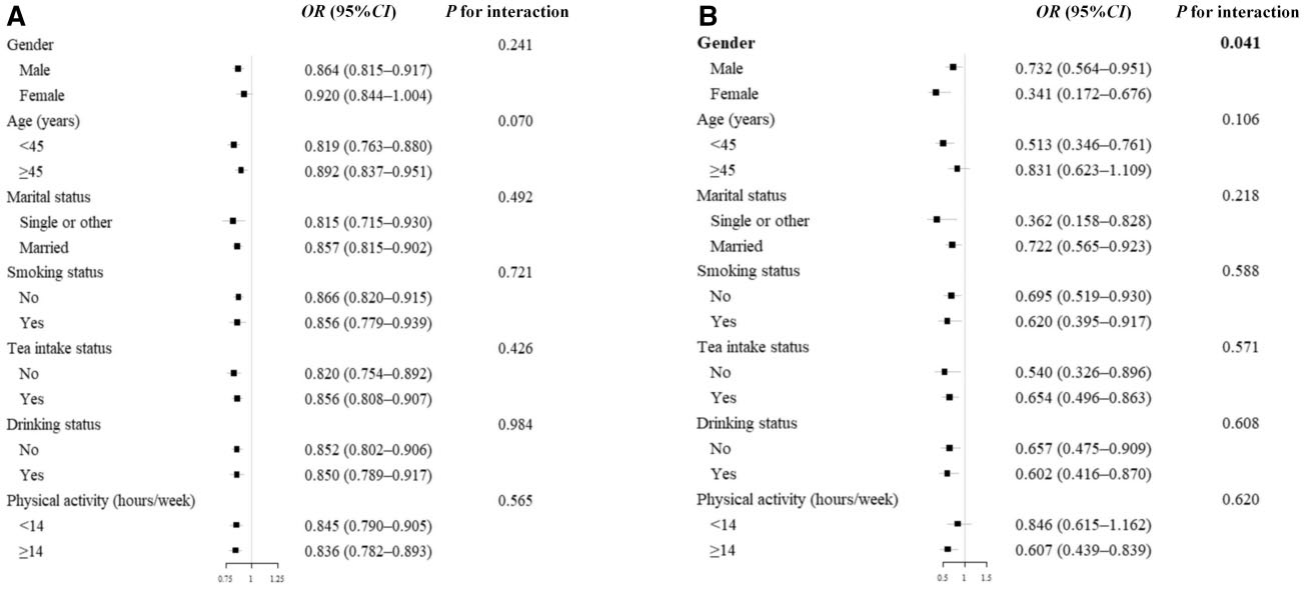
Forest plot of the association between dietary and MAFLD risk in stratified analyses. (A) Forest plot of OR of milk. (B) Forest plot of OR of tubers.

In subgroups analyses, associations of milk and tubers with MAFLD remained consistent across all subgroups of interest (Figure 3). In particular, there was a significant interactive effect between tubers and gender (*P *=* *0.041). Women (OR: 0.341, 95% CI: 0.172–0.676) had a significantly lower risk of MAFLD through consuming more tubers than men (OR: 0.732, 95% CI: 0.564–0.951).

## Discussion

In this cross-sectional study, we observed that MAFLD was associated with dietary components, such as beverages (soft drinks and sugar-sweetened beverages), instant noodles, milk, tubers, and vegetables. To our knowledge, this is the first cross-sectional study to investigate the association between foods and MAFLD.

Our results are in line with previous studies showing that beverages were positively correlated with fatty liver disease [22–26]. In 2007, a cross-sectional analysis based on interview, biochemical analysis, and radiological examination of Israeli adults (*n *=* *375) reported a correlation of NAFLD with higher consumption of meat and sugar-loaded soft drinks [22]. The beverages often contain high levels of high-fructose corn syrup, which can lead to significant elevations in both triglyceride and blood glucose levels [25]. Fructose can increase de novo lipogenesis and mitochondrial coupling, leading to oxidative stress, and promote fat accumulation in the liver [27, 28]. Another important component of added sugar in beverages is glucose, which can directly promote hepatic fat accumulation and thus induce metabolic syndrome [29].

Currently, there is no research on the association between instant noodles and MAFLD. But the high-calorie content, as well as the high concentration of fats and sodium in instant noodles [30], is known to be a contributing factor to an increased risk of metabolic disease [31]. A cross-sectional study in South Korea found that the frequency of instant noodle consumption was positively correlated with plasma triglyceride levels, diastolic blood pressure, and fasting glucose levels [32]. Heavy metals and polycyclic aromatic hydrocarbons in instant noodles also increase human health risks [33–35].

Apart from the above, we found that milk, tubers, and vegetables were inversely associated with MAFLD. In a prospective cohort study on Korean adults aged 40–69 years, compared with participants who did not consume dairy products, men and women aged ≥50 years who consumed milk, as well as women aged ≥50 years who consumed cheese, showed a significantly lower risk of developing NAFLD [36]. Another Korean cohort study also found that dairy consumption was associated with lower incidences of metabolic syndrome and hyperglycemia in middle-aged and older Korean adults [37]. There are several mechanisms that might explain a relationship between milk and reduced risk of MAFLD. Dairy protein from milk may have a synergistic effect with exercise in suppressing NAFLD and preventing sarcopenia, which is a known risk factor for NAFLD [38, 39]. In addition, insulin resistance is closely associated with NAFLD. The population-based prospective Coronary Artery Risk Development in Young Adults study found an inverse association between frequency of dairy intake and development of insulin resistance syndrome [40].

Existing work suggests that tubers and vegetables could have a beneficial effect on NAFLD [41–43]. The mechanism underlying the potential beneficial effects of tubers and vegetables consumption on the risk of NAFLD has not been fully elucidated. However, this can be explained by the fact that eating fruits and vegetables rich in various polyphenols and carotenoids can reduce food energy density. Tubers and vegetables are foods high in fiber, bioactive phytochemicals, and antioxidants. Phytochemicals and antioxidants can decrease lipid peroxidation and oxidative DNA damage, and prevent liver steatosis due to their anti-inflammatory properties [44–47]. Fibers also play their role by maintaining the concentrations of glucose, insulin, and free fatty acids [48, 49]. Tubers and vegetables generally have a high water content and low energy density, which reduce the overall energy density of foods and enhance satiation while reducing calorie intake [50, 51].

Currently, excessive caloric intake and nutritional patterns rich in saturated fat, carbohydrates, and sugar-sweetened beverages have all been implicated in the development of obesity and liver steatosis [52]. High BMI as a result of these poor diets may also have had a potential impact on the study results. Studies have confirmed that changing BMI through diet can improve liver function and hepatic steatosis [53, 54]. Hence, it is strongly recommended that individuals diagnosed with MAFLD or those at a high risk of developing it should make an effort to avoid or minimize their consumption of unhealthy food choices. Instead, adopting a health-promoting diet like the Mediterranean diet, or other similar diets such as a vegetarian or Dietary Approaches to Stop Hypertension diet, would be beneficial. By doing so, it is possible to slow down or modify the progression of hepatic steatosis and its natural course.

This study has limitations. On the one hand, the analysis was based on data from cross-sectional surveys that cannot be used to infer causal relationships. On the other hand, the information used in this study was obtained by retrospective self-reporting and thus the possibility of biases in the accuracy of recall information could not be eliminated.

## Conclusions

In conclusion, the risk of MAFLD may be reduced by increasing the intake of milk, tubers and vegetables, and reducing the consumption of beverages (soft drinks and sugary drinks) and instant noodles.

## Supplementary Material

goad054_Supplementary_Data
