# Supplementary material for: Association of food intake with a risk of metabolic dysfunction-associated fatty liver disease: a cross-sectional study
Source: Gastroenterol Rep (Oxf). 2023 Sep 11;11:goad054. doi: 10.1093/gastro/goad054 (PMC10495696; doi:10.1093/gastro/goad054)
Supplement: goad054_Supplementary_Data [file goad054_supplementary_data.docx]

**Supplementary Table 1.** Univariable and multivariable analysis of associations between foods and MAFLD

| Food species (per 50 g/day increase) | Crude OR (95% CI) | *P* | Adjusted OR (95% CI)* | *P* |
| --- | --- | --- | --- | --- |
| Smoked foods | 0.571 (0.242–1.349) | 0.201 | 0.533 (0.217–1.314) | 0.172 |
| Baked goods | **0.565 (0.385–0.829)** | **0.003** | 0.746 (0.509–1.094) | 0.133 |
| Pickled foods | 0.954 (0.841–1.082) | 0.464 | 0.930 (0.813–1.064) | 0.291 |
| Fried foods | 1.053 (0.912–1.216) | 0.483 | 1.053 (0.896–1.238) | 0.528 |
| Legumes | 1.022 (0.858–1.217) | 0.809 | 1.072 (0.887–1.295) | 0.470 |
| Egg | 0.920 (0.738–1.148) | 0.461 | 0.976 (0.794–1.199) | 0.815 |
| Nut | 0.967 (0.909–1.029) | 0.293 | 0.981 (0.918–1.047) | 0.557 |
| Beverage (soft drinks and sugar-sweetened beverages) | 1.233 (0.878–1.731) | 0.226 | **1.571 (1.075–2.294)** | **0.019** |
| Candy | 0.611 (0.297–1.257) | 0.181 | 0.926 (0.452–1.897) | 0.833 |
| Fruit | 0.926 (0.845–1.015) | 0.101 | **0.844 (0.772–0.922)** | **<0.001** |
| Coarse cereals | 0.838 (0.687–1.023) | 0.083 | 0.921 (0.749–1.132) | 0.435 |
| Milk | **0.841 (0.802–0.881)** | **<0.001** | **0.906 (0.861–0.953)** | **<0.001** |
| Tubers | **0.658 (0.518–0.835)** | **0.001** | **0.694 (0.542–0.888)** | **0.004** |
| Vegetables | 0.983 (0.958–1.009) | 0.199 | 0.979 (0.952–1.006) | 0.129 |
| Instant noodles | 1.373 (0.880–2.143) | 0.162 | 1.365 (0.879–2.121) | 0.166 |
| Red meat | **1.076 (****1.006**–**1.150)** | **0.032** | 1.026 (0.950–1.108) | 0.511 |
| Seafood | 1.055 (0.962–1.158) | 0.256 | 0.980 (0.885–1.086) | 0.698 |
| Poultry | 1.087 (0.887–1.334) | 0.421 | 1.012 (0.810–1.265) | 0.914 |

MAFLD, Metabolic associated fatty liver disease

* Adjusted by age, gender, marital status, smoking status, drinking status, tea intake status and weekly hours of physical activity

**Supplementary Table 2.** Propensity score matched univariable and multivariable analysis of associations between foods and MAFLD

| Food species (per 50 g/day increase) | Crude OR (95% CI) | *P* | Adjusted OR (95% CI)* | *P* |
| --- | --- | --- | --- | --- |
| Smoked foods | 0.591 (0.218–1.608) | 0.303 | 0.587 (0.184–1.871) | 0.587 |
| Baked goods | 0.779 (0.562–1.080) | 0.135 | 0.803 (0.565–1.142) | 0.222 |
| Pickled foods | 0.942 (0.816–1.088) | 0.418 | 0.962 (0.832–1.113) | 0.605 |
| Fried foods | 1.466 (0.945–2.275) | 0.088 | 1.362 (0.916–2.026) | 0.127 |
| Legumes | 0.990 (0.802–1.222) | 0.927 | 1.046 (0.843–1.298) | 0.680 |
| Egg | 0.887 (0.704–1.118) | 0.311 | 0.900 (0.710–1.141) | 0.384 |
| Nut | 0.948 (0.881–1.019) | 0.148 | 0.958 (0.890–1.031) | 0.254 |
| Beverage (soft drinks and sugar-sweetened beverages) | 1.203 (0.798–1.813) | 0.378 | 1.105 (0.728–1.676) | 0.639 |
| Candy | 1.119 (0.463–2.699) | 0.803 | 1.085 (0.445–2.647) | 0.857 |
| Fruit | 0.938 (0.853–1.031) | 0.184 | 0.951 (0.863–1.048) | 0.307 |
| Coarse cereals | 0.949 (0.764–1.180) | 0.640 | 0.998 (0.798–1.247) | 0.983 |
| Milk | **0.856 (0.811–0.904)** | **<0.001** | **0.866 (0.819–0.915)** | **<0.001** |
| Tubers | **0.747 (0.577–0.966)** | **0.026** | **0.762 (0.587–0.990)** | **0.042** |
| Vegetables | 0.972 (0.944–1.002) | 0.067 | 0.978 (0.949–1.009) | 0.157 |
| Instant noodles | **4.967 (1.965–12.551)** | **0.001** | **3.962 (1.562–10.048)** | **0.004** |
| Red meat | 1.023 (0.939–1.114) | 0.602 | 0.993 (0.910–1.083) | 0.873 |
| Seafood | 1.050 (0.931–1.184) | 0.430 | 1.011 (0.893–1.143) | 0.867 |
| Poultry | 1.055 (0.818–1.361) | 0.680 | 1.015 (0.783–1.316) | 0.909 |

MAFLD, Metabolic dysfunction-associated fatty liver disease

* Adjusted by smoking status, drinking status, tea intake status and weekly hours of physical activity
